# Bulk Fill Composites Have Similar Performance to Conventional Dental Composites

**DOI:** 10.3390/ijms21145136

**Published:** 2020-07-20

**Authors:** Håvard J. Haugen, Danijela Marovic, Matej Par, Minh Khai Le Thieu, Janne E. Reseland, Gaute Floer Johnsen

**Affiliations:** 1 Department of Biomaterials, Institute of Clinical Dentistry, University of Oslo, PO Box 1109 Blindern, NO-0376 Oslo, Norway; h.j.haugen@odont.uio.no (H.J.H.); m.k.l.thieu@odont.uio.no (M.K.L.T.); j.e.reseland@odont.uio.no (J.E.R.); g.f.johnsen@odont.uio.no (G.F.J.); 2 Department of Endodontics and Restorative Dentistry, School of Dental Medicine, University of Zagreb, Gunduliceva 5, 10000 Zagreb, Croatia; mpar@inet.hr

**Keywords:** bulk fill, composite resin, restorative materials

## Abstract

The aim of the study was to perform comprehensive characterization of two commonly used bulk fill composite materials (SDR Flow (SDR) and Filtek™ Bulk Fill Flowable Restorative (FBF) and one conventional composite material (Tetric EvoCeram; TEC). Eleven parameters were examined: flexural strength (FS), flexural modulus (FM), degree of conversion, depth of cure, polymerisation shrinkage (PS), filler particle morphology, filler mass fraction, Vickers hardness, surface roughness following simulated toothbrush abrasion, monomer elution, and cytotoxic reaction of human gingival fibroblasts, osteoblasts, and cancer cells. The degree of conversion and depth of cure were the highest for SDR, followed by FBF and TEC, but there was no difference in PS between them. FS was higher for bulk fill materials, while their FM and hardness were lower than those of TEC. Surface roughness decreased in the order TEC→SDR→FBF. Bisphenol A-glycidyl methacrylate (BisGMA) and urethane dimethacrylate were found in TEC and FBF eluates, while SDR released BisGMA and triethylene glycol dimethacrylate. Conditioned media accumulated for 24 h from FBF and TEC were cytotoxic to primary human osteoblasts. Compared to the conventional composite, the tested bulk fill materials performed equally or better in most of the tests, except for their hardness, elastic modulus, and biocompatibility with osteoblasts.

## 1. Introduction

The reason for the prevalence of composite resins as restorative materials in dentistry is that, in addition to providing superior aesthetics, they allow long manipulation and, unlike amalgams of glass ionomers, they provide unlimited working time for placing a filling. This is possible due to photoinitiators that start a polymerisation reaction immediately after activation with blue light of wavelengths in the range of 400–500 nm. Glass fillers and silica ensure the resistance of the material to various flexural, tensile, and shear forces generated by mastication. The disadvantage of photocurable composites is that light scattering on the filler–resin interface restricts the penetration of light into areas deeper than 2 mm, leaving them under-polymerised. This necessitates an incremental placement technique, rendering the placement of conventional composites a complex procedure with numerous opportunities to make a mistake [1].

Bulk fill composite resins were designed to facilitate this procedure, since they enable dentists to use thicker layers of composite filling materials, in increments of 4–5 mm [2,3]. This has been achieved by several compositional modifications, one of them being the utilisation of a lower filler volume load and larger filler particles with accordingly smaller specific surface areas, thus ensuring less light scattering and better light transmission through the restoration material. Another compositional adjustment was to select resin monomers and fillers with closely matched refractive indices in unpolymerised material, and to incorporate additional highly reactive photoinitiators. High-molecular-weight monomers are incorporated into some bulk fill materials to diminish polymerisation shrinkage (PS) and mitigate the negative effects of PS stress, which results in a combination of deep curing and adequate marginal adaptation [4,5]. However, it seems that some manufacturers, rather than radically modifying a material’s chemical composition, have simply reduced the quantity of pigments and used larger filler particles to enhance its translucency [6].

Bulk fill composites are becoming increasingly popular among practitioners, which has made them the subject of numerous scientific studies. Although they are commonly categorised into high-viscosity (sculptable, full-body) and low-viscosity (flowable, base) materials, different strategies are used and combined to achieve application in 4-mm-thick layers. Some low-viscosity bulk fills have a filler load comparable to high-viscosity materials, while some high-viscosity materials have mechanical properties similar to low-viscosity bulk fills [7]; therefore, it is not surprising that most studies have identified a lack of uniformity of the materials’ properties for both material groups (low-viscosity or high-viscosity), consequently precluding generalised clinical applications [8]. The properties are, in fact, highly material-specific [6,9,10]. 

High variability in testing conditions, specimen dimensions, and curing protocols, combined with inadequate proprietary information concerning the materials’ compositions, also introduce an element of speculation into scientific enquiry. Therefore, the numerical values of the same properties for identical materials from different studies cannot be directly compared if all the parameters are not the same and correct conclusions cannot be drawn. Instead of testing the individual properties of a large number of materials, a comprehensive characterisation of a limited number of selected materials may be more beneficial for gaining insight into their relative strengths and weaknesses. Extensive investigations on the same material batch, with the same curing conditions and with the standardized set of tests assure confident and factual reasoning. 

The aim of this study was to conduct a thorough investigation of the important physical, chemical, mechanical, and biological properties of two commonly used bulk fill composites compared to a conventional composite. The selected materials are most frequently used in public dental surgeries in Europe. Eleven different parameters were examined: flexural strength (FS), flexural modulus (FM), degree of conversion, depth of cure, PS, elemental analysis, filler morphology, Vickers hardness, surface roughness following simulated toothbrush abrasion, monomer elution, and the cytotoxic reaction of three human cell types. The null-hypothesis was that there would be no differences between the tested materials with regard to any of the examined parameters.

## 2. Results

**Figure 1:**
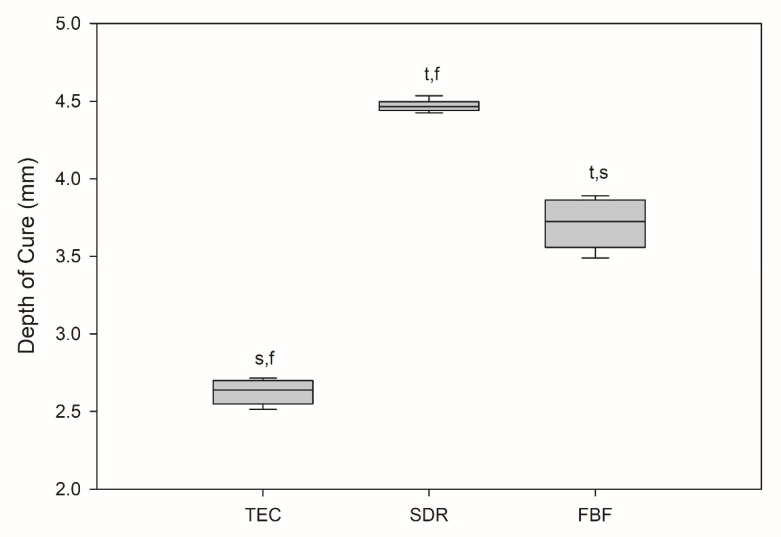
Depth of cure (mm). Box plots are shown with median in solid line, whiskers represent maximum/minimum values. t = *p* < 0.05 vs. TEC, s = *p* < 0.05 vs. SDR, f = *p* < 0.05 vs. FBF (*n* = 6).

All the results are expressed as medians, unless otherwise stated. The depth of cure was statistically lowest for TEC at 2.64 mm, as shown in Figure 1. 

**Figure 2:**
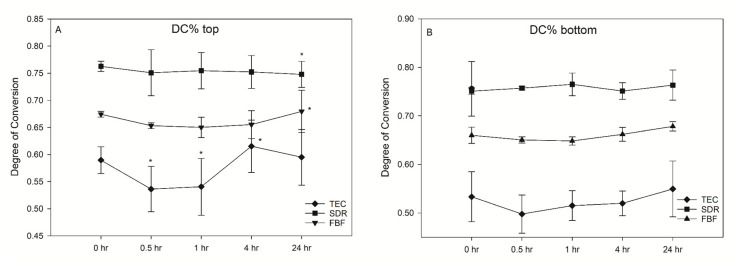
Change in the degree of conversion (DC%) over time (mean (SD)) on the top of samples (**A**) and bottom of samples (**B**) (*n* = 6).

SDR exceeded the target curing depth at 4.47 mm, while FBF reached 3.72 mm, thereby not meeting the 4 mm requirement. Degree of conversion was the highest for SDR, followed by FBF and TEC, at all measurement time points and at both the tops and bottoms of the samples, except SDR at 0.5 h versus FBF at 24 h, and SDR at 24 h versus FBF at 24 h at the bottom of the specimen (Figure 2). At the top, the DC ranged from 53.6–61.5% for TEC, to 74.8–76.3% for SDR, and 65.0–68.0% for FBF. For the bottom DC, the values ranged from 49.3–57.1% for TEC, to 75.0–77.4% for SDR, and 64.4–68.0% for FBF. 

There was no significant post-cure increase in DC for either of the tested materials. Interestingly, a decrease in DC was noted for TEC at 0.5 h (53.6%) and 1 h (54.1%) after light curing, compared to the 4 h (61.5%) at the top surface of the specimen. This effect was not present at the bottom surface.

**Figure 3:**
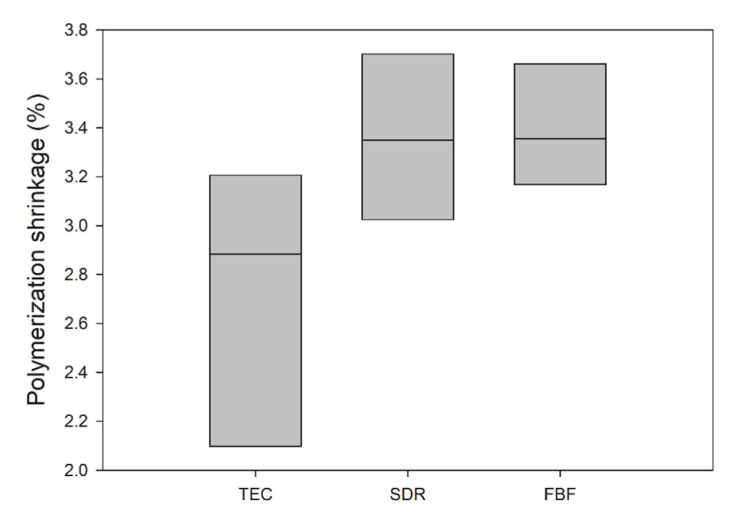
Comparison of polymerization shrinkage (solid line: median). Inter-quartile range is not shown due to *n* = 3. No significance was found between the groups (*n* = 3).

Polymerisation shrinkage is depicted in Figure 3. The mean value of PS for TEC was 2.73 ± 0.57 vol%, 3.36 ± 0.34 vol% for SDR, and 3.39 ± 0.25 vol% for FBF. There was no statistically significant difference between the materials.

Filler mass fraction was determined by thermal gravimetry (Table 1). The total inorganic mass fraction, expressed as mean ± SD, for TEC was 73 ± 0.7%, which was statistically higher than both SDR (67.6 ± 0.1%) and FBF (63.5 ± 0.8%).

**Figure 4:**
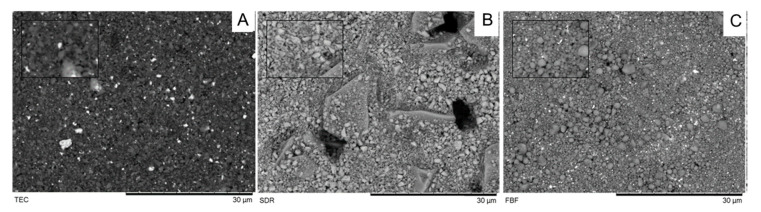
SEM images of investigated composites observed at 1000× and 3000× (small box) (**A**: TEC, **B**: SDR; **C**: FBF).

Filler morphology observed under the SEM is shown in Figure 4. Irregularly shaped filler particles of 1–3 µm were observed in TEC specimens. By contrast, FBF was mainly characterised by spherical fillers of various sizes, up to 5 µm. SDR had the most distinctive filler morphology, with irregularly shaped filler particles of two sizes. Larger filler particles were 10–20 µm in size and surrounded by smaller 1–2 µm particles.

**Figure 5:**
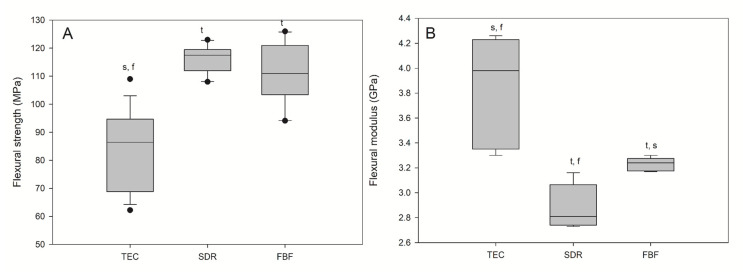
Flexural strength (**A**) and flexural modulus (**B**) of tested materials. Box plots are shown with median in solid line, whiskers represent maximum/minimum values, and solid dots represent 5th/95th percentiles. Statistically significant difference: t = *p* < 0.05 vs. TEC, s = *p* < 0.05 vs. SDR, f = *p* < 0.05 vs. FBF (*n* = 5).

Flexural strength and flexural modulus are shown in Figure 5. The lowest FS (96.80 MPa) and the highest FM (3.98 GPa) were observed for the reference material, TEC. The bulk fill materials acted similarly in terms of FS and FM, showing significantly higher FS and significantly lower FM compared to TEC. All the materials met the ISO requirement of average flexural strengths of 80 and 50 MPa for sculptable and flowable composites, respectively.

**Figure 6:**
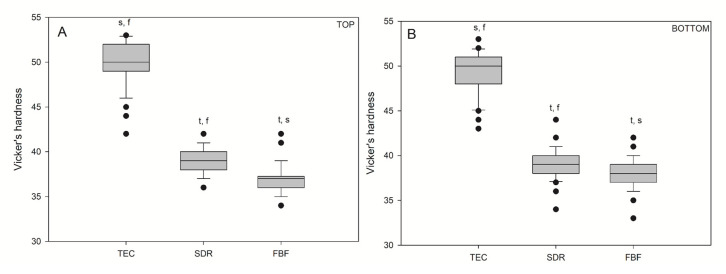
Vickers hardness measured on the top (**A**) and bottom (**B**) of the samples. Box plots are shown with median in solid line, whiskers represent maximum/minimum values, and solid dots represent 5th/95th percentiles. Statistically significant difference: t = *p* < 0.05 vs. TEC, s = *p* < 0.05 vs. SDR, f = *p* < 0.05 vs. FBF. No statistically significant differences were found between the top and bottom (*n* = 50).

Vickers hardness values for the bulk fill materials were significantly lower (*p* < 0.05) than those for the conventional reference composite, TEC (Figure 6), on both the top and bottom specimen surfaces. There was no difference in hardness between the top and bottom values within the same material: TEC (49.94/49.14), SDR (38.98/39.16), and FBF (36.96/37.92).

**Figure 7:**
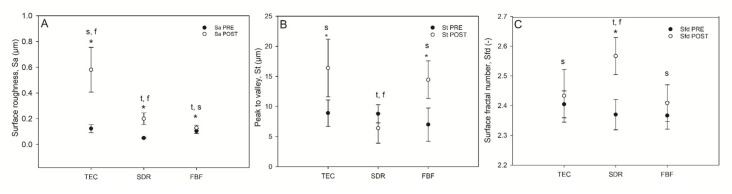
Selected surface topography parameters of composites before and after wear. Surface roughness (Sa (µm) [**A**]), top-to-bottom (St [**B**] and surface fractal dimension (**C**) of the composites pre-and post-wear. Statistically significant difference: t = *p* < 0.05 vs. TEC, s = *p* < 0.05 vs. SDR, f = *p* < 0.05 vs. FBF; asterisk (*) denotes *p* < 0.05 for pre- vs. post-wear pairwise comparisons (*n* = 9).

Selected surface topography parameters: surface roughness, total peak height, and surface fractal dimension, before and after simulated toothbrushing, are shown in Figure 7. 

**Figure 8:**
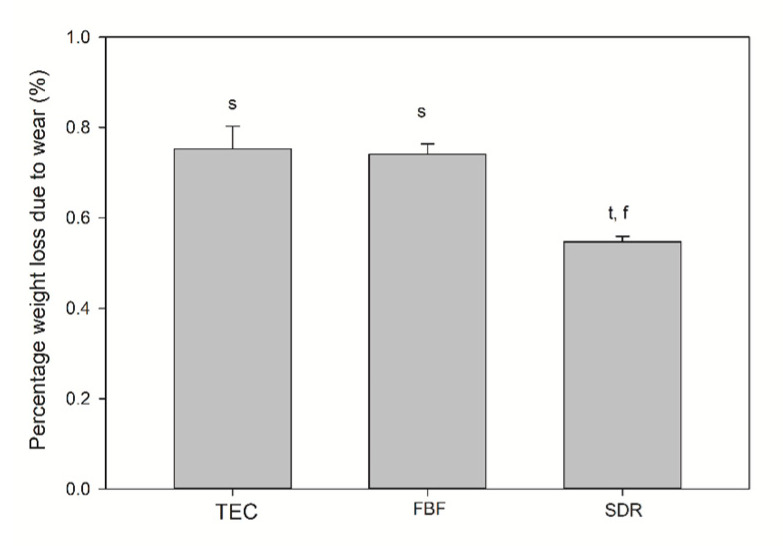
Weight loss after wear testing. Statistically significant difference: t = *p* < 0.05 vs. TEC, s = *p* < 0.05 vs. SDR, f = *p* < 0.05 vs. FBF (*n* = 5).

The TEC showed greater roughness and top-to-bottom peak height on the surface after brushing, while the SDR had a smoother surface after brushing than TEC, but a significantly lower top-to-bottom dimension and a higher surface fractal dimension number after simulated wear. There was no difference in the surface roughness of FBF before and after wear, but the surface top-to-bottom and surface fractal dimensions were higher. Figure 8 shows the weight loss after simulated toothbrushing. A significantly lower weight loss was noted for SDR (mean 0.55 ± 0.01%) compared to TEC (mean 0.75 ± 0.05%) and FBF (mean 0.74 ± 0.02%).

**Figure 9:**
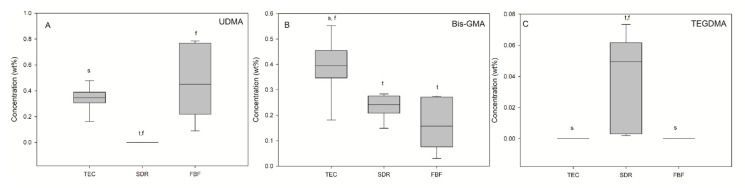
Elution of the three different monomers (**A**—UDMA, **B**—Bis-GMA, **C**—TEGDMA). Statistically significant difference: t = *p* < 0.05 vs. TEC, s = *p* < 0.05 vs. SDR, f = *p* < 0.05 vs. FBF (*n* = 9).

Monomer elution from the cured materials was detected by HPLC and the results are presented in Figure 9. UDMA (0.34 wt%) and BisGMA (0.39 wt%) were detected in elution solutions of TEC, while TEGDMA was below the detection level. SDR was the only material for which TEGDMA release (0.05 wt%) was found. Besides TEGDMA, BisGMA (0.23 wt%) was identified in SDR eluates, which was a significantly lower amount than in TEC eluates. FBF eluates also contained BisGMA (0.16 wt%) and UDMA (0.45 wt%). 

**Figure 10:**
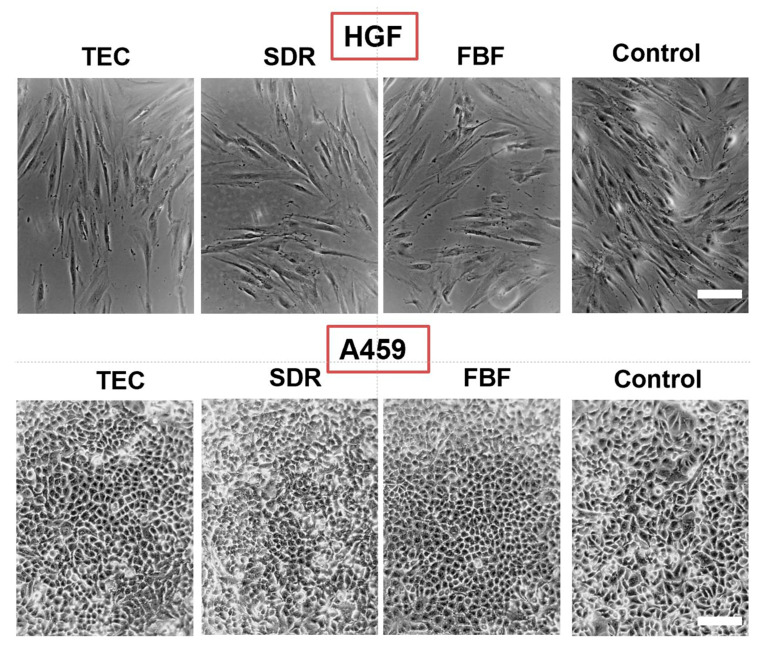
Morphology of HGF (upper panel) and A469 cells (lower panel) after 24 h incubation in conditioned media. Control are cells cultured in regular media. All images are representative phase-contrast photographs at 10× magnification and have scalebar of 10 µm.

Regarding cytotoxicity, Figure 10 shows the differences in cell morphology of HGF and A549 cells incubated in the extracts of the various materials for 24 h. In both HGF and A549 cell lines little differences were seen in the morphology of the cells cultured. 

**Figure 11:**
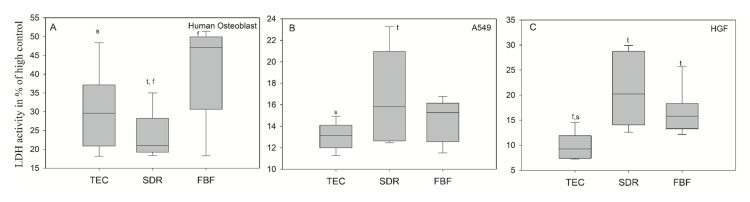
LDH activity measured from culture media of (**A**) human osteoblasts; (**B**) epithelial like cell line A549 (**B**) and (**C**) human gingival fibroblast (HGF) cells after 24 h incubation in conditioned media. Statistically significant difference: t = *p* < 0.05 vs. TEC, s = *p* < 0.05 vs. SDR, f = *p* < 0.05 vs. FBF (*n* = 6).

In Figure 11A, the extracts after 24 h of culturing for the dental composites FBF and TEC in hOB showed significantly higher LDH activity (mean values 41.34 ± 12.72% and 30.15 ± 10.54%, respectively), compared to the untreated cell control group showing cytotoxicity values greater than 30%–which is the maximum value accepted for the cytotoxicity of medical devices according to ISO-10993:5. The LDH activity in hOB was 23.48 ± 6.28% for incubation in conditioned media from SDR, and the LDH activities for A549 (Figure 11B) and HGF cells (Figure 11C) were well below 30% difference from control for all groups. SDR had significantly higher LDH values than TEC in A549 cells (mean values 16.71 ± 4.27% and 13.08 ± 1.29%, respectively), while both SDR (20.25%) and FBF (15.80%) were more cytotoxic than control material TEC (9.24%) in HGF cells. 

## 3. Discussion

Bulk fill composite resins are a diverse group of materials with highly heterogeneous features, the most distinguished one being the ability to adequately polymerise in 4-mm-thick layers. Various filler and resin modification strategies, used to facilitate adequate curing and other prerequisites, lead to the different performance of various products. The present study focused on the two most well-known and frequently purchased low-viscosity bulk fill composites in the Akershus region of Norway [11,12] and examined them using an array of mechanical, polymerisation, and biological tests. Our findings indicated that, other than for FM, Vickers hardness, and cytotoxicity on primary human osteoblasts, the tested bulk fills produced equal or better results than the conventional reference composite in all the other categories examined; thus, the null hypothesis was partially rejected.

Commonly mentioned clinical issues relating to composite restorations are secondary caries and bulk fractures, but also wear, marginal degradation, and tooth sensitivity [13,14]. It should be noted that these problems are not only due to inherent material shortcomings, but also due to patient-, tooth- and operator-related factors [15,16]; however, disregarding human factors, the material properties tested in this study are considered to be major indicators of material performance and important for the ultimate success of composite restorations. We divided these properties into photopolymerisation, mechanical, and biological properties.

### 3.1. Photopolymerisation Properties

DC is a fundamental property of composite resins and affects virtually all other mechanical properties (strength, FM, hardness, and wear), PS, and biocompatibility. A theoretically optimal 100% conversion is, however, never achieved, unfortunately being obstructed by the mobility restrictions of reactive species, due to the increasing viscosity of the polymerising network [17] and the presence of filler particles [18]. Major aspects of polymerisation occur rapidly in the first few minutes and up to 1 h after irradiation, continuing at a slower pace for up to 24 h [19]. 

Among numerous other factors, it seems that the final DC attainable under ideal curing conditions is governed by the chemical structure and type of the dimethacrylate resin, along with the photoinitiators and their concentrations [19]. SDR features a patented modified UDMA oligomer characterised by high molecular weight (848 g/mol) that also incorporates a so-called ‘polymerisation modulator’. This term signifies photoactive groups embedded in the oligomer backbone. When exposed to light, intramolecular photocleavage occurs and several radicals are formed from the oligomer. The radicals contribute to the polymerisation reaction and cross-linking and, simultaneously, the molecule adapts to polymerisation stress [20]. This mechanism allows for greater conformational flexibility during polymerisation and delays the gel point of SDR [10]. In the present study, the ATR-FTIR measurements of DC demonstrated that SDR was the best polymerised material of the tested composites, followed by FBF and TEC. These results were supported by the literature [10,21,22]. Nevertheless, one should be careful about comparing DC results for the same material obtained by different spectroscopic methods. Remarkable research by Bolaños-Carmona et al. compared FTIR, ATR-FTIR, and FT-Raman spectroscopic determination of DC for contemporary bulk fill composites, together with two different calculation methods. They concluded that different vibrational spectroscopy methods give significantly different results for the same materials tested under identical experimental conditions [23]; thus, it is best to limit the material comparisons to the same study or, at least, to the same method. 

FBF contains a proprietary Procrylat monomer, with a high molecular weight monomer similar to BisGMA, but without pendant hydroxyl groups to increase viscosity. The low viscosity of FBF is also achieved by reducing its filler content. Low filler content reduces light scattering, enabling deeper light penetration and, thus, higher DC. Some of the fillers in FBF are silanised zirconia/silica particles with a high refractive index, leading to lower light transmission compared to other low-viscosity bulk fills, including SDR [24]. This could have caused the slightly lower DC and DOC of FBF compared to SDR in the present study. 

Several papers have found a significant post-cure increase in DC for some (both high- and low-viscosity) bulk fill and conventional composites [19,21,22,25]. The present study did not show a significant post-cure DC increase, but there was a transient decrease for TEC on the top specimen surface which, after 4 h, returned to close to the initial 0 h values. However, while previous studies used dry storage of specimens, either at room- or at body-temperature, the specimens in the present study were stored in a water bath at 37 °C. This treatment was used in order to simulate the immediate exposure of the cured restoration to an aqueous environment under clinical conditions. The transient DC decrease observed for TEC after 0.5 h might have been an experimental artefact caused by the mobilisation of unreacted monomers due to specimen immersion. Since the post-cure reaction proceeded simultaneously with elution, its effect eventually surpassed the artificial DC decline, leading to a plateau of statistically similar values. In a study by Alshali et al., a post-cure increase in DC for FBF was also not present, but was detectable for SDR [22]; however, they used thin samples and relatively low irradiation (600 mW/cm^2^, 20 s). The DC data was generally characterised by highly heterogeneous variances, leading to considerable differences in the capability of multiple comparisons to detect statistically significant differences. This led to some of the multiple comparisons between different post-cure times (TEC at 0.5 h vs. TEC at 4 h, and TEC at 1 h vs. TEC at 4 h, all at the top surface), indicating statistically significant DC differences, whereas no general post-cure DC increase was identified. The inter-material comparisons at given time points showed statistically significant results in most cases, but failed to indicate significance for time points at which variances were excessively high (SDR at 24 h vs. FBF at 24 h at the top surface); therefore, the results of the multiple comparisons of the DC data must be considered in the light of highly heterogeneous variances, and the statistically significant differences identified between certain groups should be interpreted as resulting from randomly attained higher statistical power in those particular cases. 

The DOC was measured according to ISO 4049:2009 regulations, which have repeatedly been criticised in relation to bulk fill materials [3,26], since they arguably overestimate the clinically relevant curing depth of bulk fill materials. Nevertheless, this parameter is still being widely used, since it gives information about the relative differences between materials for the same curing conditions within an individual study. In the present study, 20 s curing was not sufficient for FBF to reach the 4-mm-level desired for bulk fill composites, while SDR surpassed this threshold. FBF was an A3 shade, while SDR was a universal shade. Although this difference in shades appeared to be an evident cause of the obtained results, it might be worth noting that the curing light transmission through a composite material is not necessarily related to its shade [17]. In addition to composite shades being defined differently by various manufacturers and not being directly comparable, there are also many separate parameters that independently influence light transmission through a composite. Musanje and Darvell suggested that light transmission through a composite material can be decreased by absorption of radiation (by photoinitiators and pigments), light scattering on the resin/filler interface, and changes in the composite refractive index [17]. When the exact material composition is concealed for proprietary reasons, it is challenging to predict the impact of individual constituents on the materials’ properties. Although not measured here, it is known that SDR’s translucency (and FBF’s to a lesser degree) increases during polymerisation due to changes in the refractive index of the polymerising network that approximate those of filler particles [24]. This leads to decreased light scattering, allowing a sufficient number of photons to reach the deep 4 mm level and generate initiating radicals; however, zirconia fillers in FBF seem to cause lower translucency, due to a higher filler–resin refractive index mismatch [24], with consequently lower DOC.

When using composites with identical resin matrices and varying filler loads, the PS decreases with increasing amounts of filler [27]. A higher filler load not only reduces the proportion of resin matrix in the composite, but also physically restrains the shrinkage behaviour during polymerisation. The PS of the bulk fill materials in this study approximated that of a conventional composite, although SDR had 15% higher DC than TEC, and FBF was 5% higher. For SDR, the relatively low PS was probably due to large molecules of modified UDMA (849 g/mol), with fewer reactive sites per molecule, while FBF excluded relatively small TEGDMA molecules (286 g/mol) with two reactive sites and included Procrylat (480 g/mol) as a partial replacement [28]. Our results were similar to those obtained by other studies [28,29,30,31]. 

### 3.2. Mechanical Properties

The mechanical properties tested in the present study included FS, FM, hardness, and wear. It is well known that these properties primarily relate to the filler load of the material, but also to the filler type, shape, and size. 

The tested materials contained 73 wt% of inorganic fillers for the conventional composite TEC, and 67.6 wt% and 63.5 wt% for SDR and FBF, respectively. These were the values obtained by thermogravimetry and they closely corresponded to the data provided by the manufacturers, with absolute differences in the range of 1–2%. Lower filler load values found in other studies were explained by the measurement method, depending on whether the filler weight was measured before or after the silanisation [32,33]. Leprince et al. also tested TEC by means of thermogravimetry and determined the filler fraction to be 60 wt% [33]. Although essentially the same method was used in our study, the composites in their study were heated to 900 °C, thus removing the entire organic fraction. TEC is one of the dental composites that contains pre-polymerised filler particles. The manufacturer claims that 34 wt% of pre-polymerised filler particles (which are polymerised microfilled composites, crushed and milled to obtain macrofillers from the organic matrix [34]) reduce PS and wear. In the present study, the composites were heated to 610 °C, meaning that some of the organic matrix remained—probably the organic component of the pre-polymerised fillers.

The contribution of pre-polymerised fillers to the overall hardness of the TEC is most likely small in comparison to reinforcing glass fillers. The density of the filler particles affects the hardness of composites, but the most important factor remains the filler load [33]. Even though TEC is one of the softest nanohybrid composites [35], it was no surprise that it was the hardest material in this study, since it contains 8–12.5 vol% higher filler fraction than the bulk fill composites. These results were in accordance with previous studies [26,33,36]. The influence of the filler load on the material hardness was additionally emphasised by the fact that the hardest material (TEC) was also the one with the lowest DC. This corroborated the findings of Leprince et al., who found that when samples were thoroughly polymerised (as evidenced here by statistically similar bottom and top values for DC and HV), the DC had a minor effect on hardness when comparing materials with large differences in the filler load [36]. In SDR and FBF–materials with a similar filler load–the higher DC in SDR probably contributed to its greater hardness. 

A noteworthy finding was that the FS of the bulk fill materials was higher than that of the conventional composite, especially considering the general recommendation that low-viscosity bulk fill materials should be covered with a layer of conventional composite due to their supposedly inadequate mechanical properties. The higher DC of SDR and FBF indicated that a densely crosslinked polymer network was formed, which provided strength [37]. In addition to having lower DC, TEC is at the lower end of the range of FS and FM values compared to other nanohybrid materials [33,35,38]. Again, these results were a consequence of lower filler load compared to other nanohybrids (around 60 vol%, 78 wt%) [35] and the presence of pre-polymerised fillers [39,40], but also due to the filler shape to lesser extent [33]. As shown by the SEM images, the fillers in TEC were mostly irregular in shape. Irregularly shaped filler particles allow the highest stress to be concentrated at the edges of the particles, thus creating favourable sites for crack initiation, which finally leads to lower FS. Future studies could benefit from additional analysis of the fractured specimens with 3-D microstructure reconstruction based on digital microscopy to visualize the possible low-resistance sites and filler particle agglomeration [41].

Unlike TEC, FBF contained mostly spherical fillers, allowing more uniform stress distribution during loading [33]; however, a more probable explanation for the higher FS of both FBF and SDR compared to a conventional composite is the fact that their FM is very low, due to low filler content [28]. A lower modulus means that the more elastic material will tolerate higher forces without fracturing during testing. Furthermore, our samples were immersed in water immediately after light activation, which could also affect their FS. Our FS results agreed with previously obtained results [9,33,36], but the FM results differed from those reported in previous studies. Fronza et al. reported similar results for SDR and FBF for dry-stored specimens (using biaxial flexure testing) [4], while our results were generally lower than those of Leprince et al. [36] and corresponded well to the values obtained by Randolph et al. after exposure to water or water/ethanol for an extended period [40]. 

FS and FM are clinically relevant parameters that influence materials’ resistance to fracture and the development of PS stress [42]. The FM should be close to that of dentine (12–20 GPa [36]), requiring a highly filled composite resin, while a lower FM is desirable for minimising the negative consequences of PS stress [36]. Low-modulus materials, such as SDR and FBF, showed low PS stress [43], with SDR being the material with significantly lower stress levels [28] and less simulated cuspal deflection [9]. These results were understandable, since SDR’s FM was significantly lower than that of FBF, as evident from this study. When used as a base for a high-viscosity material, the best marginal adaptation was achieved by SDR, despite its relatively high PS [44]. Clinical studies confirmed that durability class II cavities in SDR, as a base material capped by a conventional composite, equalled those of the standard incremental layering technique [45].

Due to the improved mechanical characteristics of conventional flowable composite resins, the indications for their use are expanding, from previous use as cavity liners to sole materials for direct posterior restorations [46,47]. It is clear that the low hardness of SDR and FBF precludes their use as the sole restorative materials for load-bearing areas, but their high FS and low FM might justify their use for small cervical restorations, as proclaimed by the manufacturer of FBF. For class V restorations, wear caused by toothbrushing is more relevant than occlusal wear. Wear of composite resins is mainly influenced by filler characteristics that determine the Sa and friction coefficient. Besides the filler load, shape, and hardness of filler particles, the quality of their bond to the organic matrix and the density of the polymer network determine their wear resistance [48]. The present study tested three-body abrasion by toothbrushing and determined that TEC was the material with the highest Sa, although significant differences before and after wear were found for all the materials. The high Sa of TEC could be attributed to large pre-polymerised fillers that bonded poorly to the resin matrix, thus allowing their easy dislodgement. The low FM of SDR and FBF probably gave them an advantage with regard to Sa, making them more pliable [48]. Interestingly, St values determining the peak-to-valley height were significantly higher after wear for TEC and FBF, but not for SDR. By contrast, SDR was the only material with significantly higher Sfd, meaning that SDR had lower peaks, but a denser arrangement, leading to a higher surface area. For this reason, it would be prudent to determine the bacterial adhesion and/or discolouring on the surface of SDR in future studies. Lower weight loss after wear testing for SDR compared to FBF and TEC could mean that the simulated toothbrushing action was not sufficient to cause displacement of its large fillers. The other probable explanation could be that SDR has a lower density than the other two materials. Volume loss measurements would have provided more useful information. Flowable bulk fill materials have scarcely been tested for wear. Shimokawa et al. tested 11 bulk fill and conventional materials for toothbrush wear and found a higher Sa for FBF and the successor of SDR (SDR flow+) than in the present study, but using a more abrasive toothpaste [49]. Ujiie et al. measured the occlusal wear of SDR flow+ and FBF, determining that their volume loss was greater than for conventional highly filled flowable composite resins [50]. While deep polymerisation efficiency was achieved by a reduction in filler load for SDR and FBF, this modification was not beneficial for their resistance to occlusal wear. In low-filled materials, there is greater space between the filler particles and the polymer matrix, which is more susceptible to wear [48,49]. 

### 3.3. Biological Properties

The biocompatibility of composite resins should be of the greatest importance for any material’s characterisation and evaluation. Regardless of outstanding mechanical or curing properties, if a material harms a patient or a practitioner, it is unsuitable for use. Residual unpolymerised monomers that remain chemically unbonded to polymers, but physically restrained, can be mobilised by the synergistic effects of water sorption, enzymatic degradation, occlusal load, and temperature changes in the oral cavity [51]. Commonly used BisGMA is susceptible to bacterial degradation by *Streptococcus mutans* [52]. Not only are resin monomers released, but also filler particles, silane, photoinitiators, and probably many other unidentified substances and degradation products [51]. 

In the present study, monomer release testing was conducted in the organic solvent acetone to mimic the worst-case scenario and extract the maximum number of residual monomers. In contrast, cytotoxicity tests simulated a more realistic situation within the limitations of an in vitro study. In addition, the dimensions, light curing, and preparation of the specimens used for the FS, FM, hardness, and DC tests were identical to those used for cytotoxicity tests, thus allowing us to draw direct conclusions based on the results of other tests. The specimen volume mimicked the effect of a large class II MOD filling on epithelial cells, fibroblasts, and osteoblasts, one and two days after restoration placement.

According to ISO standard 10993-5:2009 for in vitro cytotoxicity testing, samples can be divided into different cytotoxicity categories: not cytotoxic, or mildly, moderately, or severely cytotoxic. The assay measured LDH activity in the cell cultivation medium. LDH is a stable enzyme normally found in cytosol in all cells, which is quickly released to the surrounding environment when the plasma membrane is damaged. The level of LDH activity indicates the degree of damage to cells and is determined by a linked enzymatic reaction, for which the end product is a water-soluble dye detected spectrophotometrically. The LDH activity is calculated based on a positive control (high control), in which all cells are treated with detergent (=100% cell death). The assay is reliable and is used for both clinical testing (blood tests) and for in-vitro (cell medium) testing of cell death [53]. 

The most deleterious effect of composites in this study was found on osteoblasts. FBF had values close to 50%, which bordered on severe cytotoxicity, while TEC had values around 30% and could be considered moderately cytotoxic. SDR had values around 20%, which was considered to be mildly cytotoxic. None of the materials were classified as ‘non-cytotoxic’ in this test. To our best knowledge, no similar research has examined the effect on osteoblast cells, and this effect should be further investigated. We hypothesise that, in extremely deep class II cavities, residual monomers could leach out, or microscopic composite dust particles generated by polishing composite restoration could become embedded in gingival tissue and come into contact with osteoblasts. However, it is impossible to estimate the amount of material to which osteoblasts are exposed under clinical conditions. The concentrations used in the present study may have been overestimated, and the exposure time was very short (24 h). The test could be useful for comparing different materials with respect to their cytotoxic effects. 

Contrary to the adverse effect of FBF on human osteoblasts, a similar effect was not identified in the epithelial and fibroblast cell lines. Low cytotoxic effect was seen for these cell lines. Depending on the type of monomers in the composite resin and their hydrophobicity, their release might be facilitated in organic solvent or water-based solutions [54]. In this study, FBF and TEC were found to release UDMA and BisGMA and SDR to release BisGMA and TEGDMA in 7 days of exposure to a strong organic solvent, but the cells were exposed to 24 h extracts into water-based cell culture medium. BisGMA, UDMA and TEGDMA contained in all tested materials are able to cause DNA strand breaks in HGF cells [55], which could be the reason for cytotoxic reaction upon exposure to eluates.

## 4. Materials and Methods

### 4.1. Selection of Materials

The examined composite materials were the bulk fill composite materials most frequently used (according to purchased volume) in public dental health surgeries in the Akershus region of Norway [56] SDR flow (SDR; Dentsply Caulk, Milford, DE, USA) and Filtek™ Bulk Fill Flowable Restorative (FBF; 3M™ ESPE™; St. Paul, MN, USA). The conventional composite used as a reference was Tetric EvoCeram (TEC; Ivoclar Vivadent, Schaan, Liechtenstein). The composition of the materials as specified by the manufacturers is presented in Table 2. All the composites were the same shade (A3), except for SDR, which was available only in a universal shade. In all the experiments, the composites were cured with the L.E. Demetron II LED curing light (Kerr Corporation, Orange, CA, USA), selected on the basis of light intensities recommended by the different material manufacturers (FBF—40 s with 550 mW/cm^2^, 20 s with 1000 mW/cm^2^; SDR—20 s with more than 550 mW/cm^2^; and TEC—15 s with more than 800 mW/cm^2^, 10 s with 1200 mW/cm^2^). 

Identical specimens (*n* = 5) were used for the measurement of flexural strength and flexural modulus (3-point bending test), as well as for the degree of conversion test, while the same specimen design was utilized for biocompatibility testing.

### 4.2. Depth of Cure

Depth of cure (DOC) was determined according to the ISO 4049:2009 protocol [57]. Cylindrical specimens (*n* = 6), 4 mm in diameter and 10 mm high, were prepared in a stainless-steel mould. The cylindrical wells were filled with the composite paste, covered with polyester strips on both sides, and pressed with a glass plate to extrude excess material. The glass plates were removed and specimens were light cured from above for 20 s. Soft material was scraped away and the DOC (mm) was calculated by dividing the remaining specimen height by two. 

### 4.3. Degree of Conversion

Degree of double bond conversion (DC) was evaluated using attenuated total reflection Fourier transform infrared spectroscopy (ATR-FTIR; Spectrum 100; Perkin Elmer Instruments, Waltham, MA, USA). Five specimens of each material were prepared in stainless steel moulds (25 × 2 × 2 mm). The samples were light cured, for the duration specified in the instructions for use, between two transparent polyester films to avoid oxygen inhibition of polymerisation, and kept in a water bath at 37 ± 1 °C. The DC on the top and bottom of the cured test specimens was measured at the following post-cure time intervals: immediately following curing (0 h), 0.5 h, 1 h, 4 h, and 24 h. The sampling was performed at the mid-infrared range, with 2 cm^−1^ resolution and 32 scans. DC was calculated from the aliphatic (1638 cm^−1^)/aromatic (1608 cm^−1^) ratio of the spectral band intensities of the cured © and uncured (U) specimens according to the following expression: DC = (1 − C/U) × 100 (%) [58]. 

### 4.4. Polymerisation Shrinkage

PS was measured with X-ray microcomputed tomography (µCT), as previously reported [59,60]. µCT-scans were performed in dark conditions using a desktop SkyScan 1172 (Bruker, Aartselaar, Belgium). Uncured samples (*n* = 3 for each composite) with a mean surface area of 40.21 mm^2^ were mounted vertically in customised tubes. Scanning parameters were set to a 17.77 µm pixel size, using 100 kV and 100 mA x-ray sources and 500 µm Al and 38 µm Cu filters. Samples were rotated 360° around its vertical axis with a rotational step of 0.7°. Next, the composites were cured for 60 s to ensure optimal curing and the scanning was performed immediately thereafter. This method allowed for scanning of five samples simultaneously. Shrinkage was calculated based on differences in volume, calculated according to the formula developed by Sun and Lin-Gibson [59], where shrinkage is SµCT, the volume of an uncured composite is V1, and the volume of a cured composite is V2: SµCT = (V1 − V2)/V1.

### 4.5. Filler Mass Fraction

The total filler content (weight percentage, wt%) of the inorganic fillers was determined using STA 449 F3 thermogravimetric analysis apparatus (Netzsch GmbH, Selb, Germany). The mass of a substance was monitored, as a function of temperature or time, while a sample specimen was subjected to a controlled temperature procedure [32]. The composite sample was placed in an aluminium crucible (DSC/TG pan Al) and heated at a flow rate of 20 °C/min to 610 °C under nitrogen atmosphere. 

### 4.6. Morphology of Filler

The morphology of the fillers and their distribution in the matrix were examined using scanning electron microscopy (SEM; Hitachi Analytical tabletop SEM TM3030, Hitachi, Japan). The filler morphology was determined by dissolving cured composites in acetone and chloroform according to Beun et al. [61]. The specimens were cut into three pieces and evaluated from top to bottom. The specimens were gold sputtered (Sputter Coater 108 auto; Ted Pella Inc., Redding, CA, USA) before observation at two different magnifications (1000× and 3000×) (*n* = 3). 

### 4.7. Flexural Strength and Flexural Modulus

A three-point bending test was performed according to ISO 4049 standards [57] on a Zwicki (Zwick/Roell, Ulm, Germany) universal testing device with testXpert (Zwick/Roell) software. Five identical specimens from each material were prepared using stainless steel moulds with the following dimensions and permissible deviations: (25 ± 2) mm × (2.0 ± 0.1) mm × (2.0 ± 0.1) mm. The top and bottom surfaces were polymerised using six overlapping irradiations of 20 s on each side. The cured specimens, still embedded in the mould, were placed in a water bath (ISO 3696 grade 2 water (37 ± 1 °C)) for 15 min. Thereafter, a crosshead speed of 0.75 mm/min was applied in the universal testing machine until failure occurred, using a preload of 0.5 N.

The FS was calculated according to the formula σ = 3FL/2bh^2^ (MPa), where F is the maximum load (force), L is the length of the support span, b is the width, and h is the thickness of the sample. The FM was determined by E = FL^3^/4bh^3^d (GPa), where d represents the mid-span deflection of the sample corresponding to load F.

### 4.8. Vickers Hardness

Five specimens of each material were prepared. Unpolymerised material was sandwiched between two polyester strips in a steel mould (7 mm × 7 mm × 2 mm) and light cured for 20 s. Ten indentations were made on each on the top and bottom of the specimen surfaces after 24 h dry storage, at a load of 1.00 kg for 15 s using a Zwick/Roell ZVH30 microhardness tester (Zwick/Roell). 

### 4.9. Surface Topography Parameters

Surface topography parameters [(surface roughness (Sa), total peak height (St), and surface fractal number (Sfd)] were determined before and after three-body abrasion (Minimise, Buehler GmbH, Dusseldorf, Germany), using a modified toothbrush and slurry/reference toothpaste (ISO 11609:2010(E)) device [62]. 

After curing, test bodies were wet polished with 4000 grit sandpaper and surface parameters were measured at 50× objective (Nikon, Tokyo, Japan) on a profilometer (Sensofar PLμ 2300, Terrassa, Spain) (*n* = 9 per composite). Specimens were stored in constant-temperature (37 ± 1 °C) ISO 3696 grade 2 water prior to circular brushing (simulating Fone’s brushing technique) [63] with 30,000 brush cycles. The toothbrush bristle heads (Butler Gum 311, GUM, Chicago, IL, USA) were kept for 24 h in ISO 3696 grade 2 water at 37 ± 1 °C before testing. Slurry/reference toothpaste was mixed with ISO Silica (SIDENT^®^ AT25747; Evonik Industries, Hanau, Germany). After brushing, the test bodies were dried at 37 °C for 24 h before the surface parameters were measured and compared with measurements taken before abrasion.

### 4.10. Monomer Elution

Residual monomer analysis was based on the ISO 20795-1:2013(E) guidelines [64]. The amount of residual monomer was measured as a weight percentage of the organic matrix (resin). 

Cured material specimens were immediately stored in acetone for seven days prior to liquid chromatographic analysis in an Agilent 1100 high performance liquid chromatograph (HPLC; Agilent Technologies, Santa Clara, CA, USA). Chromatography was performed at ambient temperature using a Symmetry C18 column (150 mm × 150 mm, 5 µm particle size, 100 Å pore size) with an injection volume of 50 µL, a flow rate of 1 mL/min, eluent A (acetonitrile in H_2_O, 50:50 v/v %); and eluent B (acetonitrile). The materials were tested for various monomers, based on their composition as given by the respective manufacturers (Table 2): 2,2-bis[4-(2-hydroxy-3-methacryl-oxypropoxy)phenyl]propane (BisGMA), urethane dimethacrylate (UDMA; 1,6-bis(methacryloxy-2-ethoxycarbonylamino)-2,4,4-trimethylhexane), and triethylene glycol dimethacrylate (TEGDMA).

### 4.11. Production of Conditioned Cell Media

Twelve identical sticks for each composite type were prepared according to the method described for flexural testing. Two sticks made up one sample and six replicates were prepared for each composite type (*n* = 18). After curing, the samples were rinsed with deionised water to remove unattached filler particles that might act as confounding factors in the cytotoxicological analysis. The volume of the two composite sticks was roughly equivalent to a large mesio-occluso-distal cavity filling (Class II MOD) 23 making up 34% by volume of an average permanent human first molar crown (584 mm^3^) 24. 

The different dental composites were placed into 6.25 mL of the respective cell culture medium (specified below). The volume of cell culture medium was based on an estimated salivary flow rate per day; the total tooth secretion of saliva was assumed to be 1000 mL/day; and the total area of the oral cavity to be 214 cm^2^, with 20% of this surface being tooth surface (43 cm^2^). Supposing full dentition in an adult (32 teeth), the mean surface area exposed to saliva per day was 1.3 cm^2^. Assuming that 20% of the saliva (200 mL/day) was uniformly distributed across the total tooth surface area, the salivary flow rate amounted to 6.25 mL/day per tooth. The composite specimens were stored for 24 h at 37 °C and 5% CO_2_. The resulting extracts (denoted as 24-h extracts) were subsequently transferred to sterile microcentrifuge tubes and stored at 4 °C. All extracts were pre-warmed at 37 °C for 12 h prior to cytotoxicity testing. 

### 4.12. Biocompatibility and Cytotoxicity Analyses

The biocompatibility and cytotoxicity analyses were based on ISO 10993-5:2009(E) and ISO 7405:2008(E) standards [65,66]. The cytotoxicity and metabolic activity were assessed in cell cultures of A549 cells (a human epithelial, lung carcinoma cell line), human gingival fibroblasts (HGFs) and human osteoblasts (hOBs) after 24 h of incubation in a conditioned medium. 

The culture growth medium for the A549 cells contained DMEM low glucose GlutaMAX™ cell culture medium (Life Technologies, Carlsbad, CA, USA) supplemented with 10% foetal bovine serum (Biosera, Boussens, France), 100 U/mL penicillin, and 100 μg/mL streptomycin (Biowest, Nuaille, France). A549 cell lines were obtained from the American Type Culture Collection (ATCC, Manassas, VA, USA). 

HGFs were obtained from Provitro GmbH (Berlin, Germany). Cells were cultured under standard conditions of 37 °C and 5% CO_2_, and maintained in DMEM low glucose GlutaMAX™ cell culture medium (Life Technologies Corp., Camarillo, CA, USA) supplemented with 10% foetal bovine serum (Biosera Inc., Philippines), 100 U/mL penicillin, and 100 μg/mL streptomycin (Biowest). 

Primary human osteoblasts (hOB; Lonza, Walkersville, MD, USA) were cultured in osteoblast basal media (OBM; Lonza Group AG, Basel, Switzerland) supplemented with 10% foetal bovine serum, 0.1% GA-1000, and 0.1% ascorbic acid. 

To test the effect of the liquid extracts of the different dental composites on cell toxicity, 2 × 10^4^ cells were seeded in each well (48-well plate) and cultured with a growth medium for 72 h. Thereafter, the growth medium was changed and replaced with the liquid extracts of the dental composites (*n* = 6) for 24 h. In addition, untreated cells cultured with a culture medium (untreated cell control, *n* = 6) and cells cultured with a culture medium supplemented with Triton X-100 1% (high control, *n* = 6) were used as assay controls according to the manufacturer’s instruction (Roche Diagnostics, Mannheim, Gemany). 

Lactate dehydrogenase (LDH) activity in the culture media, after 24 h incubation with the exudates, was used as an index of cell death. LDH activity was determined spectrophotometrically after 30 min of incubation at 25 °C with 100 μL of culture and 100 μL of the reaction mixture by measuring the oxidation of NADH at 490 nm in the presence of pyruvate, according to the manufacturer’s instructions (Roche Diagnostics, Indianapolis, IN, USA).

Cell morphology of the HGF and A549 cells was visualised after cell culture for 24 h with the different extracts. Prior to imaging the cells were fixed for 15 min with 4% formaldehyde in PBS at room temperature. Representative phase-contrast images of cells were taken at 10× magnification and compared to untreated cells at the same time point (Nikon Eclipse TS100).

### 4.13. Statistics

Statistical analyses were performed using SigmaPlot 14.0 statistical software (Systat Software, San Jose, CA, USA). All tests were performed at a confidence level of 95% normality (Shapiro–Wilk tests (*p*-value for rejection 0.05)), and equal variance tests (Brown-Forsythe tests (*p*-value for rejection 0.05)) were performed prior to further statistical testing of the combined batch values. When the datasets were normally distributed, statistical comparison of the different groups was performed using one-way analysis of variance (ANOVA) test followed by *post hoc* tests for pairwise comparisons performed using Student–Newman–Keuls tests. The datasets that failed normality or equal variance testing were analysed using non-parametric Kruskal–Wallis one-way ANOVA, with multiple comparisons performed using Tukey tests. Statistical significance was determined at a probability of *p* < 0.05. Pairwise comparisons of the means were performed using a Student t-test after testing for normality. Mann-Whitney U/Wilcoxon Rank-Sum testing was used in cases of failed normality instead of a Student’s t-test. Statistical significance was determined at a value of *p* < 0.05.

## 5. Conclusions

Within the limitations of the present study, the results indicated higher DOC and DC and equal PS of the tested bulk fill composites compared to a conventional composite resin. The DOC was 4.47 mm, 3.72 mm, and 2.6 mm, while DC at the sample surface after 24 h was 74.8 ± 0.02%, 68.0 ± 0.04%, and 59.5 ± 0.05% for SDR, FBF and TEC, respectively. The PS was similar for all materials, 3.3 ± 0.2%, 3.4 ± 0.4%, and 2.7 ± 0.6%, for SDR, FBF and TEC, respectively.

The mechanical properties of the bulk fill composites were less desirable, although their FS was higher (119.0 and 119.0 MPa for SDR and FBF, respectively) than that of the conventional composite (96.8 MPa). Low FM (3.24 and 3.98 GPa for SDR and FBF, respectively) and hardness (39 and 37 HV for SDR and FBF, respectively) preclude the use of low-viscosity bulk fill composites, without additional capping with a high-viscosity material, in areas with occlusal load. Although the bulk fill composites showed satisfactory wear resistance to toothbrushing in this study, further investigations are necessary to confirm whether they could be used for restorations not involving contact with masticatory forces, such as class V cavities. 

The biocompatibility of bulk fill composites was similar to control material when in contact with epithelial cells and fibroblasts. However, due to the fact that 40% of osteoblast cells were affected by FBF, its use in deep class II cavities should be avoided, because of possible diffusion of its degradation products to osteoblast cells. Close follow-up and testing of new materials on the market should be prioritised.

## Figures and Tables

**Table 1:** Filler mass fraction measured by thermal gravimetry. Statistically significant difference compared to TEC: * *p* < 0.05 (*n* = 3).

Sample	Weight%	SD
TEC	73.0	0.7
SDR *	67.6	0.1
FBF *	63.5	0.8

**Table 2:** Composition of tested materials provided by manufacturers.

Material/(Abbreviation)	Resin	Filler	Filler wt%/vol%	Shade
Tetric EvoCeram (TEC)	BisGMA, UDMA, Ethoxylated Bis-EMA	Barium glass filler, YbF3, mixed oxide, prepolymers	75–76/53–55	A3
SDR Flow (SDR)	SDR patented urethane di-methacrylate resin, di-methacrylate resin, di-functional diluents	Barium and strontium alumino-fluoro-silicate glasses	68/45	universal
Filtek Bulk Fill Flowable Restorative (FBF)	BisGMA, BisEMA(6), Procrylat and UDMA	zirconia/silica filler, YbF3	64.5/42.5	A3

Abbreviations: BisGMA (2,2-bis[4-(2-hydroxy-3-methacryloxypropoxy)phenyl]propane), BisEMA(6) (2,2-Bis[4-methacryloxypolyethoxyphenyl)propane], Procrylat (2,2-bis[4-(3-methacryl-oxypropoxy)phenyl]propane), UDMA (urethane dimethacrylate), TEGDMA (tri[ethylene glycol] dimethacrylate); YbF_3_ (ytterbium trifluoride).

## Abbreviations

TEC	Tetric EvoCeram
SDR	SDR Flow
FBF	Filtek Bulk Fill Flowable Restorative
BisGMA	Bisphenol A-glycidyl methacrylate
UDMA	Urethane dimethacrylate
TEGDMA	Triethylene glycol dimethacrylate
DC	Degree of conversion
DOC	Depth of cure
PS	Polymerization shrinkage
FS	Flexural strength
FM	Flexural modulus
Sa	Surface roughness
St	Total peak height
Sfd	Surface fractal number
HV	Vickers hardness
HGF	Human gingival fibroblasts
hOB	Primary human osteoblasts
LDH	Lactate dehydrogenase
